# Evaluating aggregate effects of rare and common variants in the 1000 Genomes Project exon sequencing data using latent variable structural equation modeling

**DOI:** 10.1186/1753-6561-5-S9-S47

**Published:** 2011-11-29

**Authors:** NL Nock, LX Zhang

**Affiliations:** 1 Department of Epidemiology and Biostatistics, Case Western Reserve University, 2103 Cornell Road, Cleveland, OH 44106-7281, USA

## Abstract

Methods that can evaluate aggregate effects of rare and common variants are limited. Therefore, we applied a two-stage approach to evaluate aggregate gene effects in the 1000 Genomes Project data, which contain 24,487 single-nucleotide polymorphisms (SNPs) in 697 unrelated individuals from 7 populations. In stage 1, we identified potentially interesting genes (PIGs) as those having at least one SNP meeting Bonferroni correction using univariate, multiple regression models. In stage 2, we evaluate aggregate PIG effects on trait, Q1, by modeling each gene as a latent construct, which is defined by multiple common and rare variants, using the multivariate statistical framework of structural equation modeling (SEM). In stage 1, we found that PIGs varied markedly between a randomly selected replicate (replicate 137) and 100 other replicates, with the exception of *FLT1*. In stage 1, collapsing rare variants decreased false positives but increased false negatives. In stage 2, we developed a good-fitting SEM model that included all nine genes simulated to affect Q1 (FLT1, KDR, ARNT, ELAV4, FLT4, HIF1A, HIF3A, VEGFA, VEGFC) and found that *FLT1* had the largest effect on Q1 (*β*_std_ = 0.33 ± 0.05). Using replicate 137 estimates as population values, we found that the mean relative bias in the parameters (loadings, paths, residuals) and their standard errors across 100 replicates was on average, less than 5%. Our latent variable SEM approach provides a viable framework for modeling aggregate effects of rare and common variants in multiple genes, but more elegant methods are needed in stage 1 to minimize type I and type II error.

## Background

The 1000 Genomes Project is an international public-private consortium aiming to build the most detailed map of human genetic variation with the overarching goal to improve our understanding of the genetic contribution to common human diseases. Initially launched in 2008, three pilot studies have been completed to test multiple sequencing methods. Pilot Project 3 involved sequencing the coding regions (exons) of 3,205 genes in 697 individuals from 7 populations, which revealed 24,487 rare and common genetic variants. The sequencing data from Pilot Project 3 were used for Genetic Analysis Workshop 17 (GAW17), and details of this data set, including how the phenotypes were simulated, can be found in Almasy et al. [1].

Although strategies have been developed to evaluate the contribution of rare variants to disease susceptibility in nonfamilial data, including collapsing methods, which are reviewed by Dering et al. [2], approaches that evaluate the combined or aggregate effects of rare and common variants together are limited. Thus in this paper we aim to evaluate the aggregate effects of rare and common single-nucleotide polymorphisms (SNPs) in genes on the simulated quantitative trait Q1 using the Pilot Project 3 data (unrelated subjects). In stage 1 we use multiple regression methods (with and without collapsing rare variants) to identify potentially interesting genes (PIGs); in stage 2, we use a latent variable structural equation modeling (SEM) approach to evaluate aggregate effects of rare and common variants in PIGs on Q1. During our initial analyses, we were blinded to the “answers” of the simulated model. In* post hoc* analyses, we used knowledge that 39 SNPs in 9 genes, primarily in the vascular endothelial growth factor (VEGF) pathway, were simulated to be associated with Q1.

## Methods

### Data cleaning and preparation: phenotype and genotype variables

We first examined the distribution of Q1, which we arbitrarily chose from the three simulated quantitative phenotypes available (see Almasy et al. [1]), in a randomly chosen replicate (replicate 137) of the unrelated individuals from the GAW17 data using SAS, v. 9.1 (SAS Institute Inc., Cary, North Carolina). Visual inspection of histograms and quantile-quantile (Q-Q) plots and Shapiro-Wilk and Kolmogorov-Smirnov tests indicated that Q1 was essentially normally distributed. Summary statistics and Mendelian inheritance errors were evaluated using PLINK, v. 1.07 [3].

### Stage 1: statistical methods for regression-based analyses

We evaluated the association between each SNP as an additive model (0, 1, or 2 copies of the minor allele) and Q1 using linear regression models adjusted for all the covariates provided in the GAW17 data set (Age, Sex, Smoking, population [Pop1]) using PLINK, v. 1.07. In addition, we collapsed rare variants (minor allele frequency [MAF] < 0.05) in each gene using the indicator coding method [2], which assumes equal weighting of each rare variant. We also adjusted the models for population substructure using principal components (PCs). PCs were generated using the centralized scoring matrix method of Qin et al. [4,5] in MATLAB (MathWorks, Boston, Massachusetts). We adjusted models for multiple PCs and found that adjusting for 10 or 12 PCs minimized the number of false positives (see Results section, Table three, and additional details in Qin et al. [5]).

### Stage 2: statistical methods for latent variable structural equation modeling

**Figure 1:**
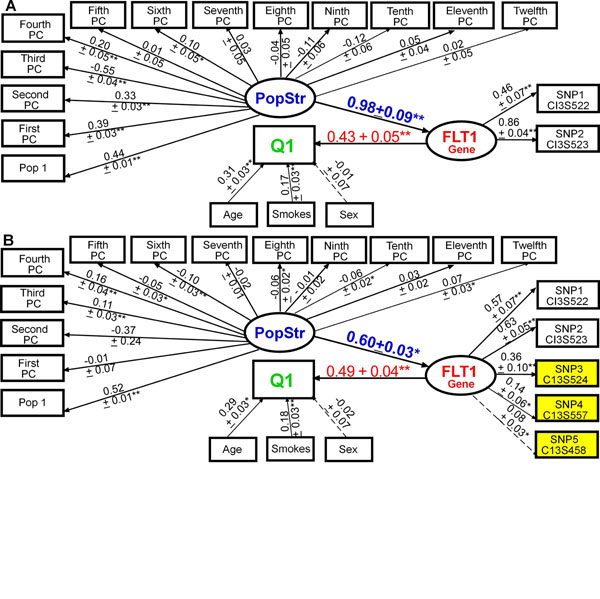
**Modeling the aggregate effects of common and rare variants in *FLT1* using latent variable structural equation modeling**. Adding rare variants (B) to the *FLT1* latent construct composed of common variants (A) improved the model fit (A: CFI = 0.90, RMSEA = 0.03, SRMR = 0.08; vs. B: CFI = 0.96, RMSEA = 0.02, SRMR = 0.05) and the variance explained in Q1 (*R^2^*: 0.36 ± 0.04 (B) vs. 0.30 ± 0.04 (A)). Standardized parameters and standard errors are shown above the arrows. Yellow, rare variant; blue, population substructure (PopStr; principal component, PC); red, gene; green, trait. * *p* ≤ 0.05; ** *p* ≤ 0.001. Residuals not shown for clarity.

Our approach for modeling multiple common variants in genes using latent constructs has been described previously [6]. Essentially, we let a latent variable (ovals in Figure 1, e.g., *FLT1*) represent the overall variation in a gene, which we formally describe by multiple SNPs (rectangles in Figure 1, e.g., C13S522) in that gene. In terms of notation, briefly, in latent variable structural equation modeling (SEM), two general submodels are used: (1) a measurement model that develops the relations (loadings; e.g., the arrow from *FLT1* to C13S522 in Figure 1) between the observed variables and the latent constructs; and (2) a structural model that develops the relations (path coefficients; e.g., the arrow from PopStr to *FLT1* in Figure 1) between the latent variables. The general form of the measurement model is:

**y** = **Λ***_y_***η** + **ε**, (1)

where **y** is the *p* × 1 vector of observed variables, **η** is the *m* × 1 vector of latent random variables, **ε** is the *p* × 1 vector of measurement errors for **y**, and **Λ***_y_* is the *p* × *n* matrix of coefficients relating **y** to **η**.

The general form of the structural model imposes constraints such that:

**η** = **Βη** + **ζ**, (2)

where **Β** is the *m* × *m* matrix of path coefficients and **ζ** is the *m* × 1 vector of errors or disturbances in the endogenous (dependent) latent variables.

The structural model can be modified by adding a *q*-dimensional vector of covariates (**x**), an *m* × *q* matrix of regression coefficients (**Γ**), and an *m*-dimensional vector of intercepts (**α**):

**η** = **α** + **Βη** + **Γx** + **ζ**. (3)

Similar to our prior work using common variants to describe overall variation in a gene [6,7], we used eigenvalues, scree plots, reliability, linkage disequilibrium plots (Haploview, v. 4.2), and association results from stage 1 to help select the most informative SNPs and define parsimonious latent gene constructs. We performed confirmatory factor analysis using a robust maximum-likelihood estimator, which provides test statistics and standard errors robust to nonnormality, using Mplus, v. 5.1 (Muthén and Muthén, Los Angeles, California), to generate and test single latent gene construct models, which included adjustment for covariates (Age, Sex, Smoking) and population structure (modeled using a latent variable defined by Pop1 and the top 12 PCs). To assess the overall model goodness-of-fit, we used the chi-square test, the comparative fit index (CFI), the root mean-square error of approximation (RMSEA), and the standardized root mean-square residual (SRMR) [8].

The chi-square test evaluates whether the covariance matrix is equal to the model-implied covariance matrix predicted by the parameters, but it is sensitive to sample size and complexity. Thus, other fit indexes, including the CFI, RMSEA, and SRMR, have been used to evaluate model fit [8]. The CFI is relatively insensitive to sample size and model complexity, and CFI ≥ 0.95 and CFI ≥ 0.90 suggest good and acceptable fit, respectively [9]. The RMSEA is less sensitive to sample size and favors more parsimonious models. An RMSEA ≤ 0.06 represents good fit, and an RMSEA ≤ 0.10 yields acceptable fit [9]. An SRMR ≤ 0.08 represents a good fit, and an SRMR < 0.10 represents an acceptable fit [8,9]. We evaluated the performance of the SEM model by calculating the mean relative bias in the parameters and their standard errors across 100 replicates (replicates 99–136 and 138–200) available in the GAW17 data [1]. All *p*-values are from two-sided tests.

## Results

Without knowledge of the underlying simulated model and using a randomly selected replicate (replicate 137), we evaluated potential associations between each SNP and trait Q1 in stage 1. We found that several genes had a least one SNP meeting or exceeding the Bonferroni-corrected level with (*p* ≤ 8.33 × 10^−6^) and without (*p* ≤ 2.04 × 10^−6^) collapsing rare variants (MAF < 0.05) (Table 1), but the most significant associations were observed with common (C13S522, C13S523) and rare (C1S3524) variants in *FLT1* (Table 2).

**Table 1:** Top potentially interests genes (PIGs) with SNPs associated with Q1 in replicate 137 of GAW17 exon sequencing data (unrelated individuals)

Gene	Chromosome	Total Number of SNPs	Distance (bp)	Crude model			Adjusted model 1^a^			Adjusted model 2^b^		
	
				Number of SNPs with *p* < 2.04 × 10^−6^	Number of SNPs with *p* < 0.10	Highest *p* (SNP)	Number of SNPs with *p* < 2.04 × 10^−6^	Number of SNPs with *p* < 0.10	Highest *p* (SNP)	Number of SNPs with *p* < 2.04 × 10^−6^	Number of SNPs with *p* < 0.10	Highest *p* (SNP)
*FLT1*	13	35	16,389	2 (C13S522 n, C13S523 n)	10 (8 n, 2 s)	3.41 × 10^−18^ (C13S523)	3 (C13S522 n, C13S523 n, C13S524 n)	11 (7 n, 4 s)	5.64 × 10^−21^ (C13S423)	2 (C13S522 n, C13S523 n)	11 (7 n, 4 s)	2.10 × 10^−11^ (C13S423)
*FADS3*	11	9	15,457	1 (C11S3071 n)	1 (1 n)	2.37 × 10^−7^ (C11S3071)	1 (C11S3071 n)	2 (1 n, 1 s)	1.33 × 10^−7^ (C11S3071)	1 (C11S3071 n)	1 (1 n)	8.68 × 10^−7^ (C11S3071)
*C5ORF25*	5	22	55,401	1 (C5S4371 n)	2 (1 n, 1 s)	3.99 × 10^−5^ (C5S4371)	1 (C5S4371 n)	2 (1 n, 1 s)	3.45 × 10^−7^ (C5S4371)	0	2 (1 n, 1 s)	4.56 × 10^−5^ (C5S4371)
*AKAP13*	15	163	223,193	1 (C15S4393 n)	10 (8 n, 2 s)	1.58 × 10^−6^ (C15S4393)	1 (C15S4393 n)	11 (8 n, 3 s)	1.37 × 10^−6^ (C15S4393)	0	14 (10 n, 4s)	3.89 × 10^−5^ (C15S4393)
*OR2T34*	1	16	495	3 (C1S11528 n, C1S11529 s, C1S11541 n)	11 (8 n, 3 s)	8.06 × 10^−9^ (C1S11541)	3 (C1S11528 n, C1S11529 s, C1S11541 n)	11 (8 n, 3 s)	2.80 × 10^−10^ (C1S11541)	0	6 (3 n, 3s)	3.28 × 10^−4^ (C1S11541)

^a^ Adjusted for Age, Sex, Smoking, Pop1.

^b^ Adjusted for Age, Sex, Smoking, Pop1 and top 12 principal components (PCs).

^c^ n=nonsynonymous SNP; s=synonymous SNP

**Table 2:** Select *>FLT1* SNPs in GAW17 exon sequencing data (replicate 137; unrelated individuals) and associations with Q1

SNP	Minor allele frequency	Crude model		Adjusted model 1^a^		Adjusted model 2^b^		Adjusted model 3^c^		Adjusted model 4^d^	
	
		*β* (SE)	*p*	*β* (SE)	*p*	*β* (SE)	*p*	*β* (SE)	*p*	*β* (SE)	*p*
**C13S320**	0.0014	1.18 (0.71)	0.0954	0.81 (0.67)	0.2227	0.78 (0.67)	0.2438	0.96 (0.65)	0.1375	0.96 (0.65)	0.1385
**C13S399**	0.0007	0.30 (1.00)	0.7676	0.13 (0.95)	0.8947	0.10 (0.95)	0.9145	−0.02 (0.92)	0.9819	−0.07(0.92)	0.9383
**C13S431**	0.0172	0.80 (0.21)	1.06 × 10^−4^	0.66 (0.19)	6.94 × 10^-4^	0.69 (0.20)	4.72 × 10^−4^	0.62 (0.22)	5.39 × 10^−3^	0.59 (0.22)	8.07 × 10^−3^
C13S458	0.0014	1.84 (0.71)	9.38 × 10^−3^	1.32 (0.67)	0.0490	1.29 (0.67)	0.0552	1.30 (0.65)	0.0465	1.32 (0.65)	0.0432
**C13S479**	0.0007	0.22 (1.00)	0.8263	0.39 (0.94)	0.6780	0.42 (0.94)	0.6546	0.29 (0.91)	0.7500	0.37 (0.91)	0.6821
**C13S505**	0.0007	−0.14 (1.00)	0.8926	0.25 (0.94)	0.7933	0.23 (0.95)	0.8094	0.34 (0.91)	0.7101	0.41 (0.92)	0.6583
**C13S514**	0.0007	1.03 (1.00)	0.3052	1.05 (0.94)	0.2660	1.08 (0.94)	0.2507	1.43 (0.91)	0.1173	1.43 (0.91)	0.1172
**C13S522**	0.0280	1.14 (0.16)	2.0 × 10^−12^	1.12 (0.15)	1.7 × 10^−13^	1.12 (0.15)	2.1 × 10^−13^	0.96 (0.16)	1.12 × 10^−9^	0.98 (0.16)	9.3 × 10^−10^
**C13S523**	0.0667	0.94 (0.11)	3.4 × 10^−18^	0.96 (0.10)	3.3 × 10^−21^	0.96 (0.10)	5.6 × 10^−21^	0.81 (0.12)	1.9 × 10^−11^	0.81 (0.12)	2.1 × 10^−11^
**C13S524**	0.0043	1.68 (0.41)	3.7 × 10^−5^	1.98 (0.38)	2.66 × 10^−7^	1.97 (0.38)	3.17 × 10^−7^	1.58 (0.38)	3.12 × 10^−5^	1.59 (0.38)	3.04 × 10^−5^
**C13S547**	0.0007	0.08 (1.00)	0.9330	0.54 (0.94)	0.5652	0.57 (0.95)	0.5457	0.70 (0.91)	0.4443	0.69 (0.91)	0.4501
C13S557	0.0072	0.92 (0.32)	3.63 × 10^−3^	0.90 (0.30)	2.78 × 10^−3^	0.89 (0.30)	2.97 × 10^−3^	0.69 (0.29)	0.0186	0.70 (0.29)	0.0171
**C13S567**	0.0007	0.43 (1.00)	0.6694	0.27 (0.94)	0.7758	0.29 (0.95)	0.7564	0.13 (0.92)	0.8837	0.15 (0.91)	0.8673

^a^ Adjusted for Age and Smoking.

^b^ Adjusted for Age, Smoking, Sex, and Pop1.

^c^ Adjusted for Age, Smoking, Sex, Pop1 and top 10 principal components (PCs).

^d^ Adjusted for Age, Smoking, Sex, Pop1 and top 12 PCs.

**Figure 2:**
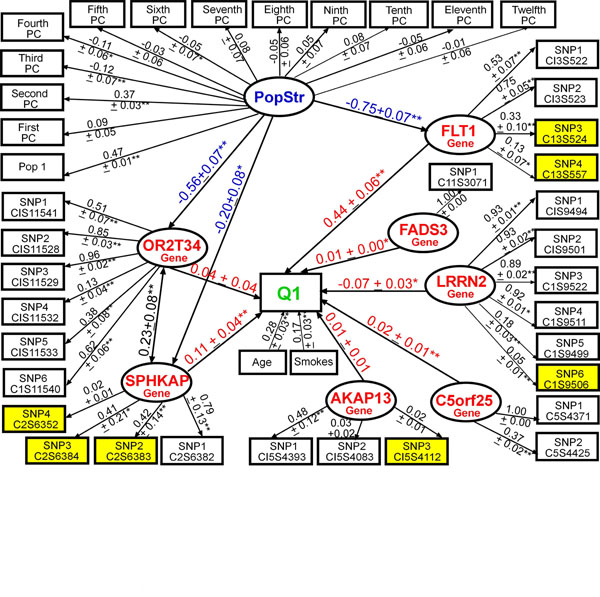
**Modeling the aggregate effects of common and rare variants in multiple potentially interesting genes (without knowledge of the GAW17 answers) using latent variable structural equation modeling.** Model of the associations between 7 putative genes (26 SNPs) and Q1 (Q1 *R*^2^ = 0.36, CFI = 0.90, RMSEA = 0.05, SRMR = 0.07). * *p* ≤ 0.05; ** *p* ≤ 0.001. Residuals not shown for clarity.

In stage 2, when building the *FLT1* construct using replicate 137, we found that adding rare variants to the common variants improved the model fit (CFI = 0.90, RMSEA = 0.03, and SRMR = 0.08 in Figure 1A vs. CFI = 0.96, RMSEA = 0.02, and SRMR = 0.05 in Figure 1B), improved construct reliability (Cronbach’s *α*: 0.40 (A) vs. 0.53 (B)), and increased the variance explained in Q1 (*R*^2^: 0.30 ± 0.04 (A) vs. 0.36 ± 0.04 (B)). In a larger SEM (Figure 2) with 6 genes (26 SNPs) and with population structure represented by a latent variable (PopStr), we found that the path coefficient of *FLT1* on Q1 (*β*_std_ = 0.49 ± 0.04) was slightly lower than that in the reduced model (Figure 1B: *β*_std_ = 0.43 ± 0.05), but *FLT1* remained the gene most strongly associated with Q1, followed by *SPHKAP*, *LRRN2*, *C5ORF25*, and *FADS3*. Genes *AKAP13* and *OR2T34* were not associated with Q1. Population structure was not significantly associated with Q1 or with genes where paths are not shown.

In post hoc analyses, we found that the list of PIGs varied markedly across replicates (99–136 and 138–200), with the exception of *FLT1*, which had at least one SNP in all 100 replicates exceeding the Bonferroni-corrected *p*-value in models adjusted for 10 or 12 PCs, and with and without rare variants collapsed. *KDR* was the next most consistent PIG, which was identified in 12 and 20 of the 100 replicates when rare variants were and were not collapsed, respectively; the results were the same when adjusting for 10 and 12 PCs.

We obtained the answers to the GAW17 simulation model to better understand the performance of our stage 1 approach and to develop a stage 2 model that would more closely reflect the simulated model. The answers revealed that 39 SNPs in 9 genes (*FLT1*, *FLT4*, *KDR*, *ARNT*, *ELAVL4*, *HIF1A*, *HIF3A*, *VEGFA*, *VEGFC*), primarily in the VEGF pathway, were simulated to be associated with Q1.

With regard to stage 1 performance, we found that the number of false-positive genes decreased with adjustment for increasing numbers of PCs. As shown in Table 3, the number of false-positive genes was lower when rare variants were collapsed (8 PCs: *μ* [mean] = 1.40, SD = 2.38; 10 PCs: *μ* = 1.25, SD = 2.35; 12 PCs: *μ* = 1.20, SD = 2.26) versus not collapsed (8 PCs: *μ* = 6.46, SD = 11.99; 10 PCs: *μ* = 5.69, SD = 11.38; 12 PCs: *μ* = 5.43, SD = 11.20). The number of true-positive and false-negative genes was similar for models adjusted for 10 and 12 PCs and when rare variants were and were not collapsed (Table 3). Relaxing multiple test criteria to *p* ≤ 1.56 × 10^−6^ (which reflects Bonferroni correction for the total number of genes) did not materially improve the number of true-positive genes when rare variants were collapsed (not shown). Although we were most interested in identifying causal genes in stage 1, we note that the number of false-positive SNPs over the 100 replicates decreased with adjustment for increasing numbers of PCs (Pop1: *μ* = 58.74, SD = 36.34; 8 PCs: *μ* = 6.68, SD = 12.38; 10 PCs: *μ* = 5.89, SD = 11.76; 12 PCs: *μ* = 5.61, SD = 11.57). The numbers of false-negative SNPs were similar when adjusting for 10 PCs (*μ* = 36.31, SD = 1.06) and 12 PCs (*μ* = 36.32, SD = 1.05) with most replicates correctly identifying *FLT1* SNPs C13S522 and C13S523 (not shown).

**Table 3:** True-positive (TP), false-positive (FP), and false-negative (FN) genes for Q1 over 100 replicates (99–136 and 138–200) in GAW17 exon sequencing data (unrelated individuals)

	Adjusted model 1^a^			Adjusted model 2^b^			Adjusted model 3^c^			Adjusted model 4^d^			Adjusted model 5^e^		
	
	TP	FP	FN	TP	FP	FN	TP	FP	FN	TP	FP	FN	TP	FP	FN
Mean	1.80	43.48	7.20	1.23	5.69	7.77	1.23	5.43	7.77	1.13	1.25	7.87	1.13	1.20	7.87
Standard deviation	0.72	26.78	0.72	0.42	11.38	0.42	0.42	11.20	0.42	0.34	2.35	0.34	0.34	2.26	0.34
Range	1–4	2–122	5–8	1–2	0–43	7–8	1–2	0–42	7–8	1–2	0–14	7–8	1–2	0–14	7–8

^a^ Adjusted for Age, Smoking, Sex, and population (Pop1).

^b^ Adjusted for Age, Smoking, Sex, Pop1 and top 10 PCs.

^c^ Adjusted for Age, Smoking, Sex, Pop1 and top 12 PCs.

^d^ Rare variants collapsed, adjusted for Age, Smoking, Sex, Pop1 and top 10 PCs.

^e^ Rare variants collapsed, adjusted for Age, Smoking, Sex, Pop1 and top 12 PCs.

**Figure 3:**
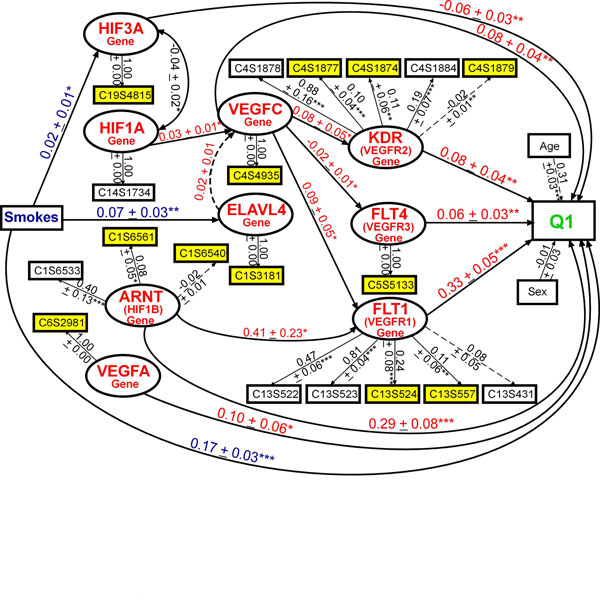
**Modeling the aggregate effects of common and rare variants in multiple genes (with knowledge of the answers) using latent variable structural equation modeling.** Model of the associations between 9 genes (19 SNPs) simulated to affect Q1 (Q1 *R*^2^ = 0.42, CFI = 0.90, RMSEA = 0.04, SRMR = 0.03). * *p* < 0.10; ** *p* ≤ 0.05; *** *p* ≤ 0.01. Residuals and paths from population structure not shown for clarity.

In regards to building the Stage 2 model, because the GAW17 answers provided only a list of the nine genes simulated to be associated with Q1, we used the pathway database of the Kyoto Encyclopedia of Genes and Genomes (KEGG) (http://www.genome.jp/kegg/pathway.html; VEGF Signaling, Cytokine-Cytokine Receptor Interaction, Pathways in Cancer) to better understand the biological relationships between the nine genes. We developed a good-fitting model (CFI = 0.90, RMSEA = 0.04, SRMR = 0.03) that included all nine genes simulated to affect Q1 (Figure 3). The variance explained in Q1 (*R*^2^ = 0.42) was greater than in prior models. *FLT1* remained the gene most strongly associated with Q1, followed by *ARNT*, *VEGFA*, *KDR*, *VEGFC*, *FLT4*, and *HIF3A*. Smoking was simulated to be associated with *KDR*, but we observed only a marginal association (*β*_std_ = 0.05 ± 0.02, *p* = 0.08; not shown) and found that Smoking was more highly associated with *HIF3A* and *ELAVL4*. Modeling all nine genes simultaneously revealed that *HIF1A* was associated with *VEGFC* but that *ELAVL4* and *HIF1A* were not associated with Q1. Removing paths designated by a dashed line (Figure 3) resulted in a slightly improved model fit (CFI = 0.91, RMSEA = 0.04, SRMR = 0.02), but the magnitude of the paths from genes to Q1 remained similar.

To evaluate the performance of the stage 2 SEM model, we used estimates from a randomly selected replicate (replicate 137) to represent population values (because the GAW17 answers did not contain standardized estimates for aggregate gene effects that we could directly compare to) and compared these population values to estimates from 100 replicates (replicates 99–136 and 138–200) available in the GAW17 data [1]. We found that the mean relative bias (MRB) in the parameters and in the standard errors across 100 replicates was 4.27% and 4.69%, respectively. The MRBs in standardized loadings and residuals were 1.47% for **Λ**, 0.09% for **ε**, and 0.57% for **ζ**; the MRBs in the standard errors (SEs) were 0.32% for **Λ**, 0.98% for **ε**, and 2.19% for **ζ**. The MRB was generally similar between common and rare variants. For example, in the *FLT1* common SNP C13S523 (MAF = 0.07) and the *FLT1* rare SNP C13S524 (MAF = 0.004), the MRB in **Λ** and the SE of **Λ** were 0.35% in C13S523, 0.62% in C13S524, and 0.33% in C13S523 vs. 0.98% in C13S524. The MRB of **ε** was 0.31% in C13S523 and 0.22% in C13S524, and the MRB of the SE of **ε** was 0.63% in C13S523 and 0.20% in C13S534. The largest bias was observed in the path coefficients (*β* = 19.9%; SE of *β* = 9.79%), which was quite severe in some cases, such as the *HIF1A* path coefficient, where the bias reached 67.94%. Interestingly, the post hoc analysis revealed that genes represented by private variants, such as *HIF1A*, that were associated with Q1 in replicate 137 were not significantly associated with Q1 in most replicates. Also, the effects of covariates (Age, Smoking, Sex, Pop1, PCs) varied markedly across replicates. Model fit, however, was generally consistent across replicates, with the average CFI, RMSEA, and RMSR being 0.91, 0.04, and 0.02, respectively.

## Discussion

In stage 1, adjusting for 10 or 12 PCs (see also Qin et al. [5]) and collapsing rare variants using the indicator coding method decreased the number of false-positive genes by about 78% (1.2 vs. 5.4), on average, but the number of false-negative genes remained high regardless of whether rare variants were collapsed or not (7.9 vs. 7.8). This is striking because we missed identifying about 87% of the simulated causal genes and correctly identified only one gene (11.1%; *FLT1*) over all 100 replicates (replicates 99–136 and 138–200). Our stage 2 SEM results were able to confirm the importance of *FLT1*, because irrespective of the other genes included in the model (i.e., the false-positive model in Figure 2 and the answer-driven model in Figure 3), the *FLT1* construct consistently had the strongest association with Q1 in replicate 137 and across all other replicates (replicates 99–136 and 138–200). We found that the MRB in the answer-driven stage 2 SEM model’s parameters and standard errors across 100 replicates was less than 5%. In addition, our stage 2 SEM model (Figure 3) revealed relationships between genes (e.g., *ARNT* and *FLT1*) and between covariates and genes (e.g., Smoking and *ELAVL4*) that were not discussed in the GAW17 answers. Thus, we believe that modeling all nine genes simultaneously together with the relevant environmental factors and population structure in a hierarchical manner that better reflects the underlying biology using latent variable SEM provides an improved understanding of each gene’s relevance in the disease pathophysiology compared to standard multiple regression methods.

## Conclusions

Our latent gene construct approach provides a viable framework for evaluating the aggregate effects of rare and common variants in multiple genes on a trait while adjusting for population substructure; however, more elegant methods are needed in stage 1 to minimize false positives and concomitantly improve identification of true-positive genes.

## Competing interests

The authors declare that they have no competing interests.

## Authors’ contributions

NLN conceived of the study, managed design and coordination, performed stage 2 analyses, and drafted the manuscript. LXZ performed data cleaning and conducted stage 1 analyses. Both authors read and approved the final manuscript.
